# Effect of taurine and acetylcysteine in attenuating microalbuminuria in type 2 diabetes

**DOI:** 10.4103/0971-4065.42349

**Published:** 2008-04

**Authors:** V. Viswanathan, M. B. Nair, P. Tilak

**Affiliations:** Diabetes Research Centre (WHO Collaborating Centre for Research, Education and Training in Diabetes), Chennai, Tamil Nadu, India

Sir,

It is shown that glomerular damage in diabetes can be prevented or at least attenuated by supplementation with taurine and *N*-acetylcysteine.^1^ Diet supplemented with taurine was found to reduce the morphological damage in experimental models with diabetes. Hence, this study was conducted to determine the benefits of treatment with combination of taurine (500 mg) and acetylcysteine (150 mg) (Nefrosave™, Fourrts (India) Laboratories Pvt. Limited) in reversal of microalbuminuria in type 2 diabetic subjects and to evaluate its potential in augmenting the renoprotective action of angiotensin-converting enzyme inhibitor (ACEI) or angiotensin receptor blocker (ARB).

The institutional ethics committee approved the study, and written informed consent was obtained from patients. Among the 41 well-controlled type 2 diabetic patients with microalbuminuria, 31 patients were given a combination of taurine and *N*-acetylcysteine along with an ACEI/ARB (treatment group) and 10 patients were treated with an ACEI/ARB alone (control group). Male:female ratio was 27:14. Both the treatment arms received similar doses of 5 mg of ACEI and 50 mg of ARB. Fasting plasma glucose, glycosylated hemoglobin triglycerides, cholesterol, urinary albumin/creatinine ratio (UACR), and serum transforming growth factor (sTGF-b1) were determined at baseline and at the end of 3 months of treatment period. The UACR was determined by immunoturbidimetry and sTGF-b1 was analyzed by sandwich enzyme linked immunosorbent assay.

There was no significant difference between the treatment group and the control group with respect to baseline parameters like age (treatment group: 59 ± 7, control group: 60 ± 12 years), duration of diabetes (treatment group: 14 ± 7, control group: 17 ± 5 years), and weight (treatment group: 70 ± 14, control group: 69 ± 11 kg). At follow-up, significant reduction in diastolic blood pressure (DBP) (baseline: 82 ± 08 vs. follow-up: 78 ± 05 mmHg, *P* = 0.021), UACR (baseline: 85 ± 59 vs. follow-up: 45 ± 25 mg/mg creatinine, *P* = 0.001), and sTGF-b1 (baseline: 18.3 ± 12.4 vs. follow-up: 13.2 ± 9.9, *P* = 0.002) were seen in the treatment group. No such significant changes were noted in the control group [DBP - baseline: 79 ± 7 vs. follow-up: 79 ± 6 mmHg, *P* = 0.2; UACR - baseline: 61 ± 29 vs. follow-up: 92 ± 52 mg/mg creatinine, *P* = 0.2; and sTGF-β1 - baseline: 20 ± 4.2 vs. follow-up: 19.7 ± 8.1, *P* = 0.6)]. Along with the above parameters, glomerular filtration rate (GFR) was also estimated using Cockroft Gault Equation.^2^ However, it was found that there was some increase in the GFR in the control group (baseline: 81.9 ± 23.9, follow-up: 95.5 ± 26.3; *P* = 0.2) compared with the treatment group (baseline: 84.0 ± 26.2, follow-up: 88.1 ± 30.3; *P* = 0.6) but the increase was not statistically significant.

This prospective study showed that taurine in combination with *N*-acetylcysteine was useful in attenuating UACR and sTGF-β1 levels in microalbuminuric type 2 diabetic patients. The benefits of taurine therapy on kidney function and blood pressure are noteworthy and may be useful in preventing the deterioration of microalbuminuria. The limitation of the study is its small sample size. A larger cohort with longer follow-up is needed to validate the findings of this study.
